# An INS/WiFi Indoor Localization System Based on the Weighted Least Squares

**DOI:** 10.3390/s18051458

**Published:** 2018-05-06

**Authors:** Jian Chen, Gang Ou, Ao Peng, Lingxiang Zheng, Jianghong Shi

**Affiliations:** School of Information Science and Engineering, Xiamen University, Xiamen 361001, China; 23320140154353@stu.xmu.edu.cn (J.C.); ougang@xmu.edu.cn (G.O.); lxzheng@xmu.edu.cn (L.Z.); shijh@xmu.edu.cn (J.S.)

**Keywords:** INS, WiFi fingerprint, pre-processing techniques, MDTW, WLS

## Abstract

For smartphone indoor localization, an INS/WiFi hybrid localization system is proposed in this paper. Acceleration and angular velocity are used to estimate step lengths and headings. The problem with INS is that positioning errors grow with time. Using radio signal strength as a fingerprint is a widely used technology. The main problem with fingerprint matching is mismatching due to noise. Taking into account the different shortcomings and advantages, inertial sensors and WiFi from smartphones are integrated into indoor positioning. For a hybrid localization system, pre-processing techniques are used to enhance the WiFi signal quality. An inertial navigation system limits the range of WiFi matching. A Multi-dimensional Dynamic Time Warping (MDTW) is proposed to calculate the distance between the measured signals and the fingerprint in the database. A MDTW-based weighted least squares (WLS) is proposed for fusing multiple fingerprint localization results to improve positioning accuracy and robustness. Using four modes (calling, dangling, handheld and pocket), we carried out walking experiments in a corridor, a study room and a library stack room. Experimental results show that average localization accuracy for the hybrid system is about 2.03 m.

## 1. Introduction

With the increasing of smartphones, there is an increasing demand for location-based services, such as fire rescue, pedestrian tracking, advertising, and marketing. At the core of location-based services is how to determine the position of a user. For outdoor positioning, global positioning systems (e.g., GPS, BDS, Glonass and Galileo) have met users’ needs. For indoor positioning using smartphones, users still desire a real-time, continuous, low power meter-level positioning system.

With the development of Micro-Electro-Mechanical Systems (MEMS), MEMS inertial sensors have been integrated into smartphones. The main advantages of these sensors are small size, low cost, low power consumption, and prolonged continuous positioning. Inertial navigation system (INS) has been successfully applied to positioning [1,2,3,4]. By integrating the acceleration and angular velocity, the position of a user is estimated. However, due to temperature drift and manufacturing errors within the inertial sensor, over time, the cumulative errors make it difficult for a single inertial sensor to provide prolonged positioning.

Wireless Access Points (APs) have been widely installed in classrooms, shopping malls, libraries, and all kinds of places of activity. Using WiFi signals for indoor localization is a very popular technology [5]. WiFi positioning models are mainly divided into attenuation models [6,7] and fingerprint matching models [5]. According to the relationship between the position of a user and the Received Signal Strength (RSS), the WiFi attenuation model is used to estimate the location of a user. Commonly used techniques are time difference of arrival [8], angle of arrival [9], and time of arrival [10]. In [7], light fidelity was used to calibrate the relevant coefficients of attenuation models when a user keeps moving inside a building. However, for complex indoor environments, many objects (e.g., walls , ground, obstacles and users) cause multipath effects [11]. Therefore, it is difficult for the attenuation model to provide reliable indoor positioning accuracy. The WiFi fingerprint matching model is another technique for estimating the location of a user. When a user collects WiFi signals, RSSs, with wireless fingerprint signals in the database, are used to calculate the distance. The fingerprint position corresponding to the shortest distance is taken as the estimated position of the user. *k*-nearest neighbor and weighted *k*-nearest neighbor are two commonly used techniques [12]. Compared with the attenuation model, it is unnecessary for the fingerprint matching model to know the AP position in advance. Signals from different WiFi chips and changes in the indoor environment lead to fingerprint mismatches. Thus, to improve indoor positioning accuracy and robustness, we propose an INS/WiFi indoor localization system using a smartphone.

However, there are some problems with the INS/WiFi hybrid system. First, the wireless signal is easily disturbed by multipath effects in complex indoor environments, resulting in a serious decline of some RSSs. Unreliable signals increase the fingerprint mismatches. Second, considering the capacity of a smartphone battery, most phone manufacturers strictly limit the WiFi sampling frequencies. In addition, the instability of the WiFi chip and the difference between the different antennas result in instability of the sampling time. Low sampling frequency increases the positioning errors. Third, traditional Dynamic Time Warping (DTW) is often used to calculate the distance for one-dimensional time series signals. Due to multiple APs in an indoor environment, smartphones collect multi-dimensional signals. Finally, multipath effects, a change in the number of APs and pedestrian activity easily cause mismatches in the radio signal fingerprints.

Taking advantage of an existing infrastructure, we propose an INS/WiFi hybrid positioning system. The integrated technology overcomes the cumulative errors in the INS and reduces the mismatching of the fingerprint. The proposed method must collect fingerprints in advance and construct a <position, RSS> database. More specifically, the main innovation points are as follows:

(1) To improve the quality of unprocessed signals from smartphones, pre-processing mechanisms, including a threshold-based detection mechanism and an adaptive interpolation mechanism, are developed . When signal strength is lower than a predefined threshold, a threshold-based detection mechanism removes the unreliable signals. An adaptive interpolation mechanism automatically inserts different wireless signals according to the sampling frequency of the smartphone.
(2) We adopt the sequence matching method, which is different from the point-to-point fingerprint matching method. To deal with multi-dimensional signals, we propose a MDTW method based on the traditional DTW. Considering the relationship between the MDTW distance and positioning error, we propose a MDTW-based WLS to reduce position error and improve robustness.
(3) Using calling, dangling, handheld and pocket motion gestures, we performed experiments in three scenarios at a local university. Experimental results reveal a positioning accuracy of 2.03 m.

The rest of the paper is organized as follows: Related work is introduced in Section 2. The system model is introduced in Section 3. Walking experiments to test different scenes are discussed in Section 4. The conclusion and future work are presented in Section 5.

## 2. Related Work

In the past decade, there have been many studies on indoor positioning. Many existing technologies rely on their sensors for indoor positioning such as inertial sensors [13,14]. The main advantage is that it is free from infrastructure deployment and is relatively inexpensive. Zheng et al. [14] designed inertial sensors and a barometer to estimate the 3D indoor position of a user. To obtain accurate positioning results, a Kalman filter with zero velocity update was used to reduce nonlinear errors in inertial navigation. However, the cumulative error of inertial sensors increases infinitely over time. In addition, the integral operation expands the cumulative error. Based on the special building layout, Borenstein et al. designed the Heuristic Drift Elimination (HDE) algorithm for personal tracking systems [15]. When a person walks in a structured man-made building, the walking heading is divided into four dominant directions. The HDE algorithm gradually adjusts the headings to the closest dominant direction. The HDE algorithm is applied only to buildings with rectangular shapes. Yu et al. proposed an INS and map integrated system using auxiliary particle filter (APF) for indoor pedestrian localization [16]. When the particles cross the wall, their weights are set to zero. By a re-sampling method, no crossing-wall particles are close to the real pedestrian location. Experimental results validate the performance of the integrated system. The performance of APF depends on the number of particles and the construction of the map. Another problem is that the map of the structure of the building involves privacy.

The use of RSS as fingerprints for indoor positioning is a popular technology. Earlier work such as RADAR [17] using WiFi fingerprints achieved a positioning accuracy of 3–5 m. However, WiFi signal is easily affected by the indoor complex environment, causing fluctuations in RSS. Li et al. [18] presented a WiFi-assisted magnetic algorithm using smartphones to solve indoor localization. Using WiFi results to limit the search range of magnetic fields reduces position errors. The WiFi-assisted magnetic system achieved indoor positioning accuracy with root mean square of 4.2–4.5 m. Li et al. [19] designed a dead-reckoning/WiFi fingerprinting/magnetic matching integration system for indoor localization. A three-level quality-control mechanism is used to improve the performance of the fusion system. In mechanism 1, several techniques are used to filter out the blunders in the measured readings. In mechanism 2, a threshold-based technique is designed to set the weight of the WiFi positioning results. In mechanism 3, DR/WiFi positioning results are used to limit the magnetic field matching space. The improved fusion system reduces the root mean square of the position errors in the range of 13.3% to 55.2%. When using fingerprints in the WiFi and magnetic fields, to increase the computational complexity of the smartphone, two fingerprint databases are needed. To reduce the time-consuming and labor-consuming burden of fingerprint acquisition, support vector regression was designed to increase WiFi-based positioning resolution without increasing the site-survey effort [20]. A two-layer EKF/APF integrated system was designed to fuse INS data and WiFi data [21]. However, APF consumes extraordinary time to select the particles, which makes the real-time positioning of smartphones very difficult.

Least squares has been successfully applied in indoor positioning. Zhuang et al. [22] designed a weighted nonlinear least squares technique using a transmission model to estimate the WiFi propagation parameters and the position of the user. When multipath effects are considered, it is difficult for the transmission model to obtain reliable, high accuracy indoor positioning. WLS based on received signal strength measurements was used for wireless local area network indoor positioning system [23]. The least squares weighting coefficient is constructed by assuming free space for the wireless signal transmission model.

Inspired by the above work, this paper proposes to use WLS to fuse INS and WiFi positioning results. The error model using WLS improves positioning accuracy by automatically adjusting the weighting coefficients. To some extent, the hybrid system of this paper is more robust.

## 3. System Model

**Figure 1:**
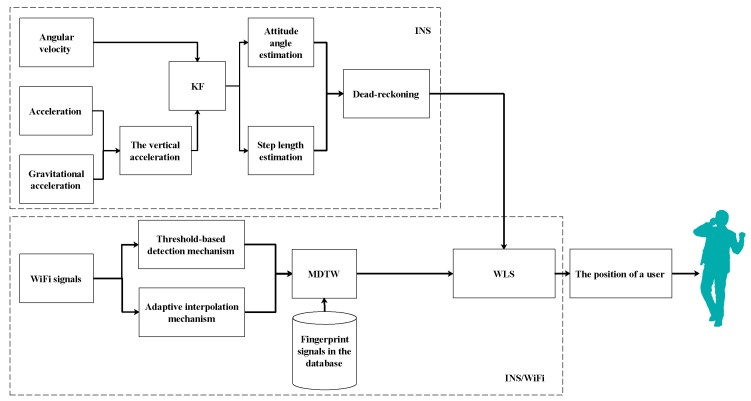
System model.

Figure 1 shows the architecture of an indoor hybrid localization system including INS module and INS/WiFi module. In INS module, the vertical acceleration is extracted by acceleration and gravitational acceleration. The Kalman filter (KF) is used to reduce the drifts from the vertical acceleration when the vertical zero velocity is detected. The user’s heading and step length can be obtained based on attitude angle model and step length model. Dead-reckoning calculates the user’s position. In INS/WiFi module, the fingerprint matching model is triggered when the smartphone receives WiFi signals. Pre-processing mechanisms (including threshold-based detection mechanism and adaptive interpolation mechanism) are designed to enhance signal quality. MDTW is used to calculate the distance between the measured signals and wireless fingerprints. Finally, to reduce position error and improve robustness, we design a MDTW-based WLS.

### 3.1. INS

INS consists of an attitude angle estimation model and a step length model. The attitude angle estimation model obtains the direction of a user by integrating angular velocity. The step length model obtains the step length of a user by integrating vertical acceleration. Finally, the location of a user is estimated based on dead-reckoning. The INS flow chart is shown as Algorithm 1.

**Algorithm 1: **INS   **Input:** Raw readings from MEMS accelerometers and gyros    1. Calculate the vertical acceleration by Equation (6).    2. Detect vertical zero velocity point.    3. **Loop INS**
   4. Compensate the IMU measurements with the current estimates of the sensor errors.    5. Update the quaternion.    6. Get attitude of the navigation system using the update quaternion.    7. Update the position and velocity estimates.    8. Use Kalman filter to estimate the zero velocity when a vertical zero velocity is checked.    9. **End INS**
   10. Calculate attitude angle by Equation (5) and step length by Equation (8).    11. Calculate a user’s position using dead-reckoning.

#### 3.1.1. Attitude Angle Estimation Model

The gyroscope collects three-axis angular velocity in the body of the coordinate system. As the attitude of the smartphone changes, the angular velocity changes accordingly. Assuming the sampling interval of the gyroscope is $T_{s}$, we calculate the three-axis angle increment $\left(\left[\Delta \theta_{k}^{x},\Delta \theta_{k}^{y},\Delta \theta_{k}^{z}\right]\right)$ at time *k* as follows:

(1) $$\begin{array}{ccc} \Delta \theta_{k}^{x} & = & \omega_{k}^{x}*T_{s}\\ \Delta \theta_{k}^{y} & = & \omega_{k}^{y}*T_{s}\\ \Delta \theta_{k}^{z} & = & \omega_{k}^{z}*T_{s} \end{array}$$

where $\left[\omega_{k}^{x},\omega_{k}^{y},\omega_{k}^{z}\right]$ denotes the gyro readings. After the angle increment is obtained, the quaternion vector $q_{k+1}=\left[q_{1},q_{2},q_{3},q_{4}\right]{|}_{k+1}$ is updated as follows [24]:

(2) $$q_{k+1}=e^{\frac{1}{2}\int_{{}_{k}}^{{}_{k+1}}M^{\prime }\left(\omega_{nb}^{b}\right)dt}\cdot q_{k}$$

where $\left[q_{1},q_{2},q_{3},q_{4}\right]{|}_{k+1}$ represents four elements of the quaternion vector $\left(q_{k+1}\right)$.

$$\Delta \Theta =\int_{{}_{k}}^{{}_{k+1}}M^{\prime }\left(\omega_{nb}^{b}\right)dt=\int_{{}_{k}}^{{}_{k+1}}\left[\begin{array}{cccc} 0 & \omega_{k}^{z} & -\omega_{k}^{y} & \omega_{k}^{x}\\ -\omega_{k}^{z} & 0 & \omega_{k}^{x} & \omega_{k}^{y}\\ \omega_{k}^{y} & -\omega_{k}^{x} & 0 & \omega_{k}^{z}\\ -\omega_{k}^{x} & -\omega_{k}^{y} & -\omega_{k}^{z} & 0 \end{array}\right]dt\approx \left[\begin{array}{cccc} 0 & \Delta \theta_{k}^{z} & -\Delta \theta_{k}^{y} & \Delta \theta_{k}^{x}\\ -\Delta \theta_{k}^{z} & 0 & \Delta \theta_{k}^{x} & \Delta \theta_{k}^{y}\\ \Delta \theta_{k}^{y} & -\Delta \theta_{k}^{x} & 0 & \Delta \theta_{k}^{z}\\ -\Delta \theta_{k}^{x} & -\Delta \theta_{k}^{y} & -\Delta \theta_{k}^{z} & 0 \end{array}\right]$$

We carry out a Talyor series expansion for Equation (2). In addition, suppose ${\left(\Delta \theta_{k}\right)}^{2}={\left(\Delta \theta_{k}^{x}\right)}^{2}+{\left(\Delta \theta_{k}^{y}\right)}^{2}+{\left(\Delta \theta_{k}^{z}\right)}^{2}$. Then, the equation is expressed as follows [24]:

(3) $$q_{k+1}=e^{\frac{1}{2}\Delta \Theta }\cdot q_{k}=\left[I+\frac{\frac{1}{2}\Delta \Theta }{1!}+\frac{{\left(\frac{1}{2}\Delta \Theta \right)}^{2}}{2!}+\cdots \right]q_{k}=\left[I\cos\frac{\Delta \theta_{k}}{2}+\Delta \Theta \frac{\sin\frac{\Delta \theta_{k}}{2}}{\Delta \theta_{k}}\right]q_{k}$$

where I denotes a 3×3 identity matrix.

When the quaternion vector is updated, the direction cosine matrix (DCM) is calculated from the corresponding quaternion vector as:

(4) $$C_{b}^{n}=\left[\begin{array}{ccc} q_{1}^{2}-q_{2}^{2}-q_{3}^{2}+q_{4}^{2} & 2q_{1}q_{2}-2q_{3}q_{4} & 2q_{1}q_{3}+2q_{2}q_{4}\\ 2q_{1}q_{2}+2q_{3}q_{4} & -q_{1}^{2}+q_{2}^{2}-q_{3}^{2}+q_{4}^{2} & 2q_{2}q_{3}-2q_{1}q_{4}\\ 2q_{1}q_{3}-2q_{2}q_{4} & 2q_{2}q_{3}+2q_{1}q_{4} & -q_{1}^{2}-q_{2}^{2}+q_{3}^{2}+q_{4}^{2} \end{array}\right]$$

When DCM is updated, the attitude angle [ϑ,ϕ,ψ] is calculated as follows [25]:

(5) $$\begin{array}{ccc} \vartheta & = & \tan^{-1}\frac{-C_{b}^{n}\left(3,1\right)}{\sqrt{{\left(C_{b}^{n}\left(3,2\right)\right)}^{2}+{\left(C_{b}^{n}\left(3,3\right)\right)}^{2}}}\\ \phi & = & \tan^{-1}\frac{C_{b}^{n}\left(3,2\right)}{C_{b}^{n}\left(3,3\right)}\\ \psi & = & \tan^{-1}\frac{C_{b}^{n}\left(2,1\right)}{C_{b}^{n}\left(1,1\right)} \end{array}$$

where ϑ, ϕ and ψ are called pitch, roll and yaw, respectively. In this paper, because the inertial positioning is a kind of relative initial point displacement, we set the initial heading in INS to zero. Another heading with respect to north is referred to [19].

#### 3.1.2. Step Length Model

The step length of a user is different for walking. A common method to estimate step length is to divide and analyze the acceleration signals. Step length models are generally categorized according to the following two groups: training-based and training-free step length models. In a training-based model, to adapt to the step lengths of different users, some experience values must be trained in advance. In [26,27,28], one or more experience values are used to optimize step length. Their main disadvantage is that different users require different training parameters, causing the training-based model to be unwelcome. The training-free model is a simple model whose step length is a constant which is obviously arbitrary because step length of a user changes. In [13], a triangulation algorithm based on a vertical acceleration is used to calculate the step length of a user. When gravitational acceleration (g) and acceleration (a) are applied, the vertical acceleration $\left(a_{z}\right)$ is calculated according to Equation (6) as follows:

(6) $$a_{z}=-\frac{(a-g)\cdot g}{\left|g\right|}$$

Vertical displacement of a smartphone is calculated by double integral vertical acceleration. During walking, *H* represents a vertical displacement in a half step, as shown in Equation (7).

(7) $$H=\int_{t_{1}}^{t_{2}}\int_{t_{1}}^{t_{2}}a_{z}dtdt$$

where $t_{1}$ and $t_{2}$ represent the beginning and end time of vertical acceleration.

**Figure 2:**
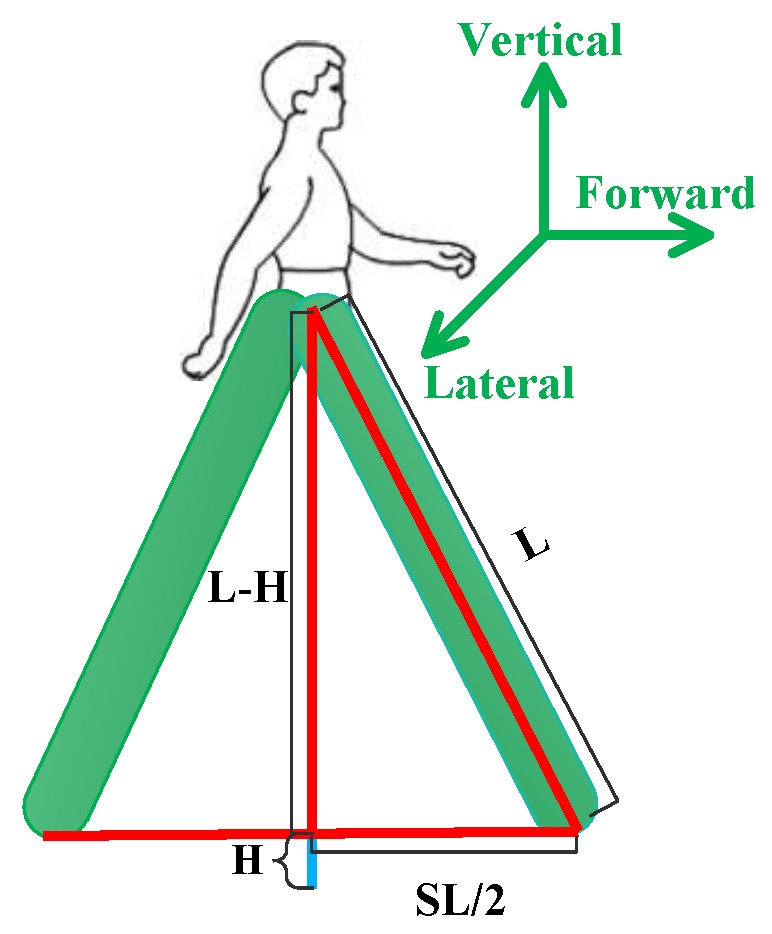
Step length model.

Figure 2 shows the relationship among step length, vertical displacement and the height of a smartphone. For a user at rest, the height of the smartphone from the ground is *L*. According to the Pythagorean theorem, the step length is calculated as follows:

(8) $$SL=2\sqrt{L^{2}-{\left(L-H\right)}^{2}}$$

#### 3.1.3. Dead-Reckoning Position

After the attitude angle and step length are estimated, the location of the user is calculated using the dead-reckoning algorithm. The dead-reckoning algorithm updates the current position, ${\left[x_{k+1},y_{k+1}\right]}^{T}$, based on the previous position, ${\left[x_{k},y_{k}\right]}^{T}$, as expressed by Equation (9).

(9) $$\left[\begin{array}{c} x_{k+1}\\ y_{k+1} \end{array}\right]=\left[\begin{array}{c} x_{k}\\ y_{k} \end{array}\right]+SL\cdot \left[\begin{array}{c} \cos\psi \\ \sin\psi \end{array}\right]$$

### 3.2. INS/WiFi Hybrid System

For the INS/WiFi hybrid system, we propose two innovations to improve indoor positioning accuracy. First, pre-processing mechanisms are used to enhance the WiFi signal quality. In mechanism 1, we use a threshold-based detection mechanism to detect WiFi signal quality and remove unreliable signals. In mechanism 2, we use an adaptive interpolation mechanism to increase WiFi frequency. Second, a MDTW-based WLS is proposed to reduce position error and improve positioning robustness.

#### 3.2.1. Pre-Processing Mechanisms

Mechanism 1. According to the wireless signal propagation model, the farther from an AP, the smaller the received signal strength. Due to multipath effects, the signal received by smartphones is seriously disturbed. A very weak RSS can easily increase the distance between the measured signals and the fingerprint. If the signal intensity involved in fingerprint matching is too low, the fingerprint mismatch increases. Therefore, to remove unreliable RSSs, we propose a threshold-based detection mechanism. When the signal strengths are lower than a predefined threshold, the unreliable RSSs are removed.

Mechanism 2. Smartphones consume much electricity when scanning WiFi signals. Considering the limited battery capacity of smartphones, many phone manufacturers have restricted the frequency of WiFi scanning. The sampling frequency for different smartphones may differ from phone to phone. Using two smartphones, we conducted WiFi signal sampling experiments. We found that the sampling frequencies of Mi 5 and Nexus 5 are 0.125 Hz and 0.5 Hz, respectively. An effective strategy to save electricity during continuous positioning is to reduce the frequency of WiFi signal sampling. However, a low sampling frequency results in increased position errors. Another problem is that the sampling frequency is unstable because of the influence of the smart antenna, WiFi chip and program software. Suppose the original sampling frequency of the smartphones is $f_{2}$. Considering computational complexity and positioning accuracy, the sampling frequency is set to $f_{1}$ after adaptive interpolation mechanism is performed. Then, the number of times required for interpolation between two adjacent WiFi signals is defined as follows:

(10) $$N=floor(\frac{f_{1}}{f_{2}})$$

where floor(a) rounds the elements of *a* to the nearest integer less than or equal to *a*.

A simple linear interpolation method is used to update the WiFi fingerprint. We assume the current and previous signals from the same AP at different position are $RSS_{2}$ and $RSS_{1}$, respectively. The adaptive interpolation mechanism is calculated as follows:

(11) $$RSS_{i}=\frac{N-i}{N}*RSS_{1}+\frac{i}{N}*RSS_{2}$$

where *i* denotes the ith interpolation signal. The adaptive interpolation mechanism, which adjusts the number of interpolation points according to the original sampling frequency, effectively balances the calculation burden and positioning accuracy.

#### 3.2.2. MDTW-Based WLS

In WiFi fingerprint positioning, single point matching is a simple technique to calculate the distance between the measured signal and the fingerprint in the database. By matching similar signals in the database, single point matching is used to estimate the location of a user. Because of the noise in the collected wireless signals, it is easy for single point matching to cause mismatches.

Compared with single point matching, sequence matching matches by using signals from multiple sampling points and uses historical information. The result of sequence matching is determined by all sequence points. Even if there is a partial point mismatch, the sequence matching may be correctly matched. Therefore, compared with single point matching, the mismatch of sequence matching will be reduced.

DTW is an algorithm to measure the similarity between two likely unequal temporal sequences. DTW either extends or shortens the sequence of measurements to reach the same length as the reference sequence. DTW has been applied to temporal sequences of video [29], audio [30], and graphics data [31]. One well-known application is automatic speech recognition, which copes with different speaking speeds [32]. In the indoor positioning system, DTW is often used to calculate the distance between two one-dimensional signals [33,34]. For WiFi fingerprint matching, each AP signal represents one-dimensional information. Multi-dimensional signals are collected because of the existence of multiple APs. Therefore, WiFi fingerprint sequence matching is a multi-dimensional fingerprint matching. MDTW has been successfully applied in gesture recognition [35]. Accordingly, we use a MDTW to calculate the distance between the collected wireless signals and the database fingerprints. Assume that RSS(k,i) represents the measured wireless signal from the kth AP and the ith sequence. ${\mathrm{RSS}}_{\mathrm{db}}\left(k,j\right)$ represents the wireless signal from the kth AP and the jth sequence in the database. The computational procedure for MDTW is shown in Algorithm 2. Unlike the traditional DTW, we replace ${\left(\mathrm{RSS}\left(k,i\right)-{\mathrm{RSS}}_{\mathrm{db}}\left(k,j\right)\right)}^{2}$ with $\sqrt{\sum_{k=1}^{p}{\left(\mathrm{RSS}\left(k,i\right)-{\mathrm{RSS}}_{\mathrm{db}}\left(k,j\right)\right)}^{2}}$ to calculate the distance among pairs of values in RSS and ${\mathrm{RSS}}_{\mathrm{db}}$.

**Algorithm 2:** MDTW  **Input:** The measured signal RSS with length *n* and fingerprint signal ${\mathrm{RSS}}_{\mathrm{db}}$ with length *m*.   1. Let d denote the distance among pairs of values in RSS and ${\mathrm{RSS}}_{\mathrm{db}}$.   2. **for** i = 1 **to**
*n*
**do**
  3. **for** j = 1 **to**
*m*
**do**
  4.  Normalize each dimension of RSS and ${\mathrm{RSS}}_{\mathrm{db}}$ separately.   5.  d(i,j) = $\sqrt{\sum_{k=1}^{p}{\left(\mathrm{RSS}\left(k,i\right)-{\mathrm{RSS}}_{\mathrm{db}}\left(k,j\right)\right)}^{2}}$.   6. **End for**
  7.**End for**
  8.Let D denote MDTW fingerprint distance from RSS and ${\mathrm{RSS}}_{\mathrm{db}}$.   9.D(1,1)=d(1,1).   10. **for** i = 2 **to**
*n*
**do**
  11. D(i,1) = d(i,1) + D(i−1,i).   12. **for** j = 2 **to**
*m*
**do**
  13.  D(1,j) = d(1,j) + D(1,j−1).   14.  D(i,j) = d(i,j) + min([D(i−1,j),D(i−1,j−1),D(i,j−1)]).   15. **End for**
  16.**End for**
  17.**Output:** The MDTW distance D.

The probability of a mismatch is lower in sequence matching than it is in single point matching. In general, the location of the smallest MDTW distance corresponds to the estimated position of the user. However, because of noise and fluctuations in the signal, mismatching cannot be eliminated completely. Least squares find the best function of the data matching by minimizing the square of the error. It has been used in indoor positioning to estimate the user’s location [22,36]. Thus, WLS is used to reduce the positioning error.

After calculating MDTW, we select *r* the MDTW distances $\left(D=\left[D_{1},D_{2},\cdots ,D_{r}\right]\right)$ and corresponding positions (${\left[{\tilde{x}}_{1},{\tilde{y}}_{1}\right]}^{T},{\left[{\tilde{x}}_{2},{\tilde{y}}_{2}\right]}^{T},\cdots ,{\left[{\tilde{x}}_{r},{\tilde{y}}_{r}\right]}^{T}$) to estimate the location of a user. Before executing WLS, we detect the credibility of the positions (${\left[{\tilde{x}}_{1},{\tilde{y}}_{1}\right]}^{T},{\left[{\tilde{x}}_{2},{\tilde{y}}_{2}\right]}^{T},\cdots ,{\left[{\tilde{x}}_{r},{\tilde{y}}_{r}\right]}^{T}$). The distance between the current position and the previous position should be limited. If the distance is greater than a threshold (e.g., several times the step length), we use dead-reckoning algorithm to update corresponding position. By linear weighting, the estimated position of the user is calculated as follows:

(12) $$\left[\begin{array}{c} \hat{x}\\ \hat{y} \end{array}\right]=\omega_{1}\left[\begin{array}{c} \tilde{x_{1}}\\ \tilde{y_{1}} \end{array}\right]+\omega_{2}\left[\begin{array}{c} \tilde{x_{2}}\\ \tilde{y_{2}} \end{array}\right]+\cdots +\omega_{i}\left[\begin{array}{c} \tilde{x_{i}}\\ \tilde{y_{i}} \end{array}\right]+\cdots +\omega_{r}\left[\begin{array}{c} \tilde{x_{r}}\\ \tilde{y_{r}} \end{array}\right]$$

where $\omega_{i}$ denotes a weighting coefficient. Equation (12) requires $\omega_{1}+\omega_{2}+\cdots +\omega_{r}=1$.

Suppose ${\left[x,y\right]}^{T}$ is the real position of the user. To minimize location error, we construct WLS objective function as follows:

(13) $$F={\left(\omega_{1}\left|\left|\left[\begin{array}{c} x\\ y \end{array}\right]-\left[\begin{array}{c} {\tilde{x}}_{1}\\ {\tilde{y}}_{1} \end{array}\right]\right|\right|\right)}^{2}+{\left(\omega_{2}\left|\left|\left[\begin{array}{c} x\\ y \end{array}\right]-\left[\begin{array}{c} {\tilde{x}}_{2}\\ {\tilde{y}}_{2} \end{array}\right]\right|\right|\right)}^{2}+\cdots +{\left(\omega_{r}\left|\left|\left[\begin{array}{c} x\\ y \end{array}\right]-\left[\begin{array}{c} {\tilde{x}}_{r}\\ {\tilde{y}}_{r} \end{array}\right]\right|\right|\right)}^{2}min$$

where ||·|| denotes the Euclidean distance.

The MDTW distance shows the difference between the measured signals and the WiFi fingerprints. The greater is the MDTW distance, the lower is the reliability of the estimated position. The position error $\Delta z_{i}$ can be expressed as $\left|\left|{\left[x,y\right]}^{T}-{\left[{\tilde{x}}_{i},{\tilde{y}}_{i}\right]}^{T}\right|\right|$. In this paper, we use exponential function to construct the relationship between the position error and the MDTW distance, which is expressed as:

(14) $$\Delta z_{i}=e^{D_{i}/\left(\sqrt{2}\sigma \right)}$$

where σ denotes a configuration parameter. Equation (13) combined with Equation (14) is then simplified as follows.

(15) $$F=\sum_{i=1}^{r}\omega_{i}^{2}\Delta z_{i}^{2}=\sum_{i=1}^{r}\omega_{i}^{2}e^{D_{i}^{2}/\left(2\sigma^{2}\right)}$$

Using the Lagrange function [37], the original objective function is rebuilt into a new objective function.

(16) $$L=\sum_{i=1}^{r}\omega_{i}^{2}e^{D_{i}^{2}/\left(2\sigma^{2}\right)}+2\lambda \left(\sum_{i=1}^{r}\omega_{i}-1\right)$$

Order $\frac{\partial L}{\partial \omega_{i}}=0$ and $\frac{\partial L}{\partial \lambda }=0$, there is

(17) $$\begin{array}{c} \left\{\begin{array}{cc} \frac{\partial L}{\partial \omega_{i}} & =2\omega_{i}e^{D_{i}^{2}/\left(2\sigma^{2}\right)}+2\lambda =0\\ \frac{\partial L}{\partial \lambda } & =2(\sum_{i=1}^{r}\omega_{i}-1)=0 \end{array}\right. \end{array}$$

By solving Equation (17), the expressions of $\omega_{i}$ and λ are obtained as follows:

(18) $$\begin{array}{c} \left\{\begin{array}{cc} \omega_{i} & =\frac{\frac{1}{e^{D_{i}^{2}}}}{\frac{1}{e^{D_{1}^{2}}}+\cdot \cdot \cdot +\frac{1}{e^{D_{r}^{2}}}}\\ \lambda & =-\frac{\frac{1}{e^{2\sigma^{2}}}}{\frac{1}{e^{D_{1}^{2}}}+\cdot \cdot \cdot +\frac{1}{e^{D_{r}^{2}}}} \end{array}\right. \end{array}$$

## 4. Experiments and Discussions

**Figure 3:**
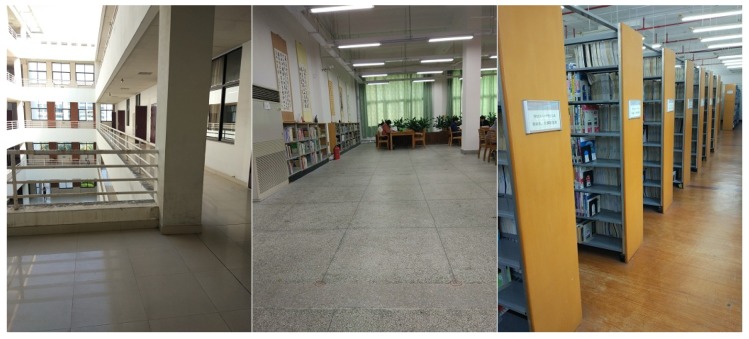
Different scenarios.

**Figure 4:**
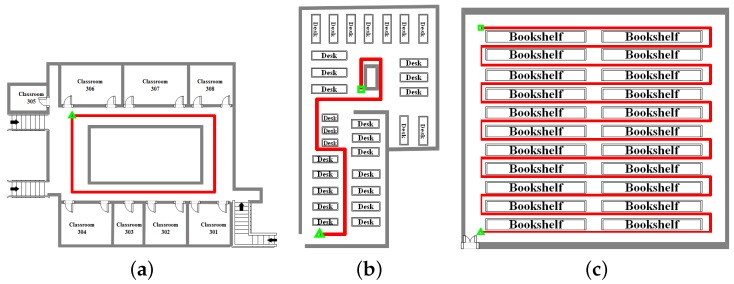
The walking trajectory: (**a**) corridor; (**b**) study room; and (**c**) library stack room.

To prove the feasibility of the hybrid algorithm, we carried out practical walking experiments in different scenarios using two smartphones (Nexus 5 for training and Mi 5 for positioning). According to ISO/IEC 18305 [38], the walking experiment scene includes a corridor, a study room and a library stack room, as shown in Figure 3. The red line in Figure 4 represents the user’s walking trajectory. Green triangle and green square represent the starting point and the ending point, respectively. It should be noted that the starting point and the ending point overlap with each other in Figure 4a. The number of APs in the three experimental scenarios are 113, 80, and 22, respectively. The walk-survey method is used to reduce the time and labor costs for fingerprint collection [18]. In the off-line phase, a surveyor carries a Nexus 5 to collect WiFi fingerprints at the experimental site based on a constant-speed assumption [18,20]. The surveyor collects RSSs along a predetermined walking path. The real surveyor trajectory is obtained with a laser distance meter. After WiFi fingerprints are collected, <position, RSS> vector is built into the database. In the experiment, six volunteers using calling, dangling, handheld, and pocket gestures tested the robustness of the hybrid system. The threshold is set to −90 dBm in a threshold-based detection mechanism, and $f_{1}$ is set to 0.5 Hz. In addition, the threshold is set to 3 m in a MDTW-based WLS. Experimental results and analysis are presented in the following sections.

### 4.1. Corridor Walking Experiment

The first experiment was carried out in the third floor corridor of a teaching building. Each classroom is equipped with some WiFi signal transmitters. With the four gestures of motion (calling, dangling, handheld, and pocket), a user walked from a starting point to an ending point. The distance was 90 m.

**Figure 5:**
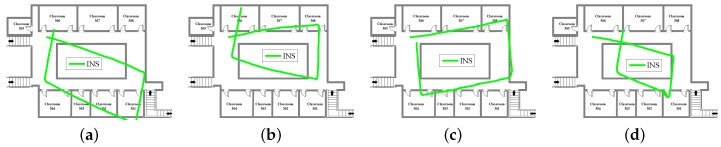
Positioning results for INS: (**a**) calling; (**b**) dangling; (**c**) handheld; and (**d**) pocket.

**Figure 6:**
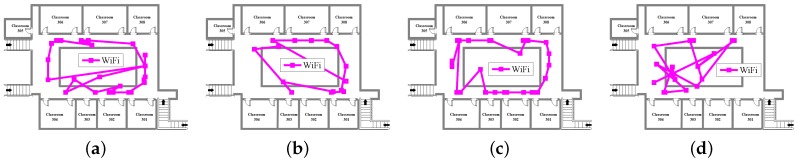
Positioning results for WiFi: (**a**) calling; (**b**) dangling; (**c**) handheld; and (**d**) pocket.

**Figure 7:**
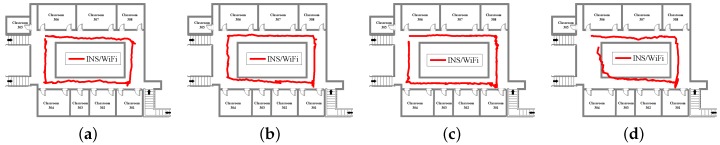
Positioning results for INS/WiFi: (**a**) calling; (**b**) dangling; (**c**) handheld; and (**d**) pocket.

Figure 5, Figure 6 and Figure 7 show INS, WiFi and INS/WiFi positioning results, respectively, for the four motion gestures. Errors in INS accumulate with time. In addition, integration enlarges the positioning errors. Accumulative errors are an unavoidable in INS. As shown in Figure 5, the positioning results for the four motion gestures verify the cumulative errors. What requires an explanation is that the cumulative errors may be briefly reduced at the turn. Indoor obstacles, movements of pedestrians, and differences in the WiFi chips cause the smartphone to receive signals that fluctuate with time. As shown in Figure 6, unstable signals cause fingerprint mismatches. Because it is difficult to improve positioning performance with a single-source system, we present an INS/WiFi hybrid positioning system to improve indoor positioning accuracy and robustness. Pre-processing mechanisms are used to enhance WiFi signal performance. Then, a MDTW-based WLS is proposed to reduce position errors. As shown in Figure 7, in regard to the four modes, the results for the hybrid positioning system are obviously better than those for INS and WiFi.

**Figure 8:**
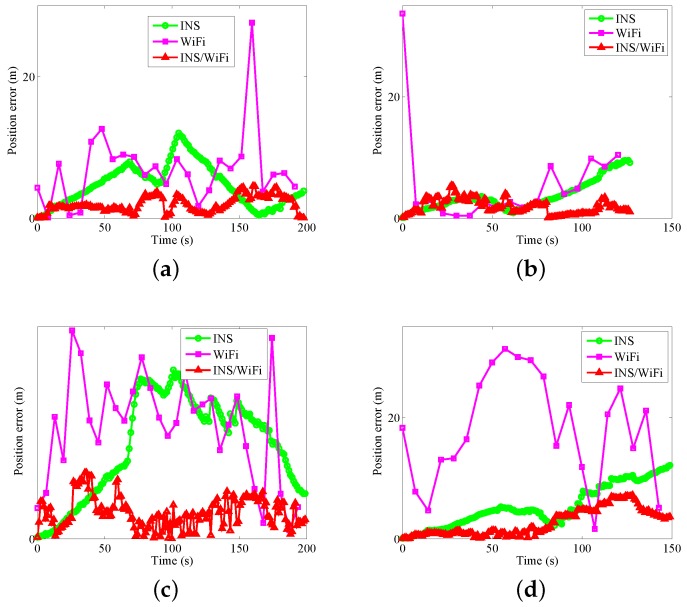
Position errors with different gestures: (**a**) calling; (**b**) dangling; (**c**) handheld; and (**d**) pocket.

**Figure 9:**
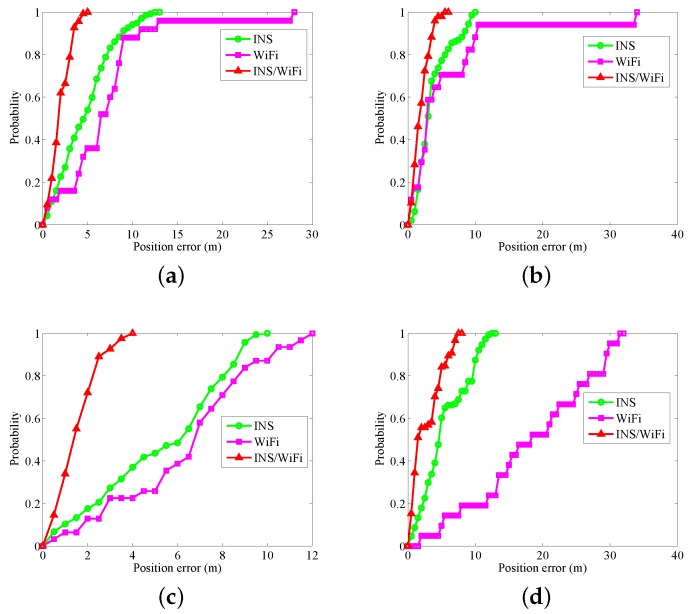
CDF with different gestures: (**a**) calling; (**b**) dangling; (**c**) handheld; and (**d**) pocket.

Figure 8 and Figure 9 show the position errors and corresponding Cumulative Distribution Function (CDF). As shown in Figure 8, in regard to the four motion gestures, usuallu, the location errors for the hybrid system are less than those for INS and WiFi. In regard to the four modes, when the positioning errors are less than three meters, the probabilities of error for INS, WiFi, and INS/WiFi are as follows: (a) for calling: 64.23%, 84%, and 21.17%, respectively (Figure 9a); (b) for dangling: 48.97%, 41.18%, and 20.69%, respectively (Figure 9b); (c) for handheld: 72.73%, 77.42%, and 7.27%, respectively (Figure 9c); and (d) for pocket: 70.2%, 95.24%, and 43.05%, respectively (Figure 9d). The positioning error for the hybrid positioning system greatly reduces.

Table 1 shows the average error, Root Mean Square Error (RMSE), maximum error, and Circular Error Probability (CEP). For the four gestures, the performances of INS and WiFi are lower than those of the hybrid system. The reason for the lower performance of INS is that the action, gesture of the smartphone, and user speed affect the readings from the inertial sensors. The reason for the lower performance of WiFi is that different phone gestures and other pedestrian movements may cause signal fluctuations. Compared with INS and WiFi, the errors of INS/WiFi are reduced as follows: 57.79% and 78.63%, respectively, for the average error; 56.53% and 78.81%, respectively, for RMSE; 51.62% and 79.97%, respectively, for maximum error; and 54.78% and 79.59% for CEP (95%), respectively.

### 4.2. Study Room Walking Experiment

Results from the corridor experiment proved that the hybrid system is superior to the single-source positioning system. Results from the study room walking experiment will prove that WLS based on MDTW is superior to RADAR [17] and MDTW [39]. The walking distance was about 110 m.

**Figure 10:**
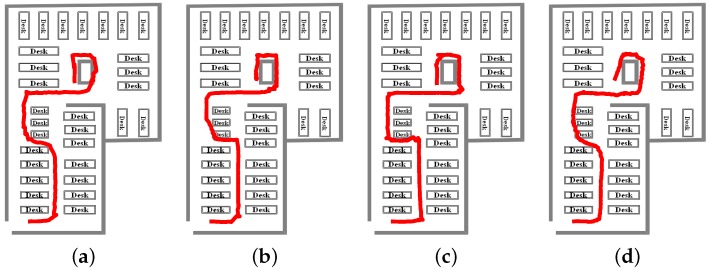
Positioning results for WLS: (**a**) calling; (**b**) dangling; (**c**) handheld; (**d**) pocket.

**Figure 11:**
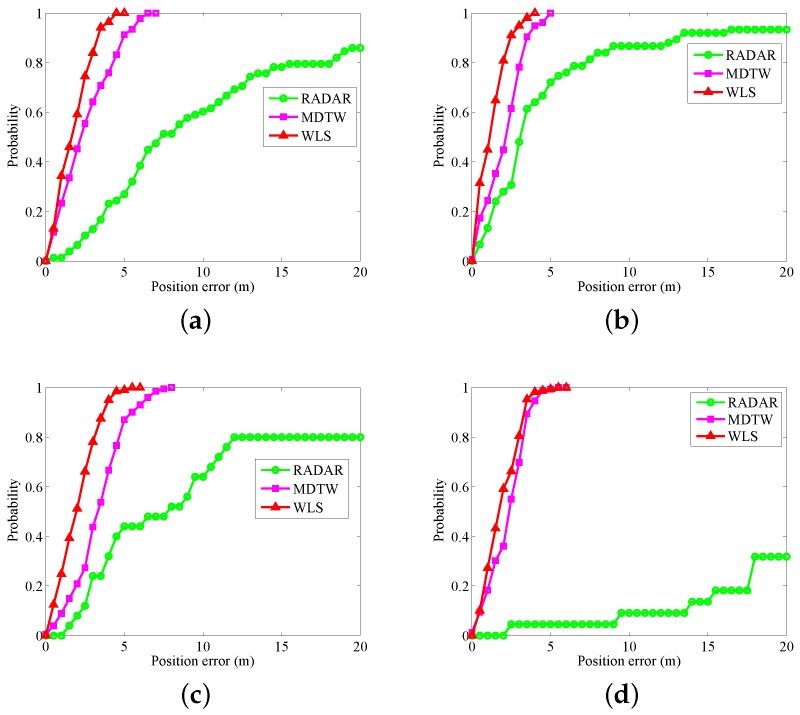
CDF for RADAR, MDTW, WLS: (**a**) calling; (**b**) dangling; (**c**) handheld; and (**d**) pocket.

Classic fingerprint positioning algorithms such as RADAR [17] use WiFi signals as position fingerprints for positioning. In [39], MDTW is used for fingerprint matching to reduce the mismatch. We use MDTW to calculate the distance between the measured signals and the WiFi fingerprint. Then, we introduce a MDTW-based WLS to improve the positioning accuracy and robustness. Figure 10 shows the positioning results of WLS. CDFs of the three algorithms are shown in Figure 11. In regard to the four modes, when the positioning errors are less than three meters, the probabilities of error for RADAR, MDTW and WLS are as follows: (a) for calling: 87.18%, 35.77%, and 16.06%, respectively (Figure 11a); (b) for dangling: 52%, 21.79%, and 5.13%, respectively (Figure 11b); (c) for handheld: 76%, 56.22%, and 21.89%, respectively (Figure 11c); (d) for pocket: 95.45%, 30.18%, and 19.53%, respectively (Figure 11d). In the four gestures, WLS obtains satisfactory positioning results.

Table 2 shows the average error, RMSE, maximum error, and CEP (95%) for RADAR, MDTW, WLS. Compared with RADAR and MDTW, the errors of WLS are reduced as follows: 87.31% and 32.91%, respectively, for the average error; 87.87% and 30.93%, respectively, for RSME; 87.83% and 21.8%, respectively, for the maximum error; and 89.08% and 28.28%, respectively, for CEP (95%). In general, the hybrid system is better than the RADAR system and the MDTW system.

### 4.3. Library Stack Room Walking Experiment

**Figure 12:**
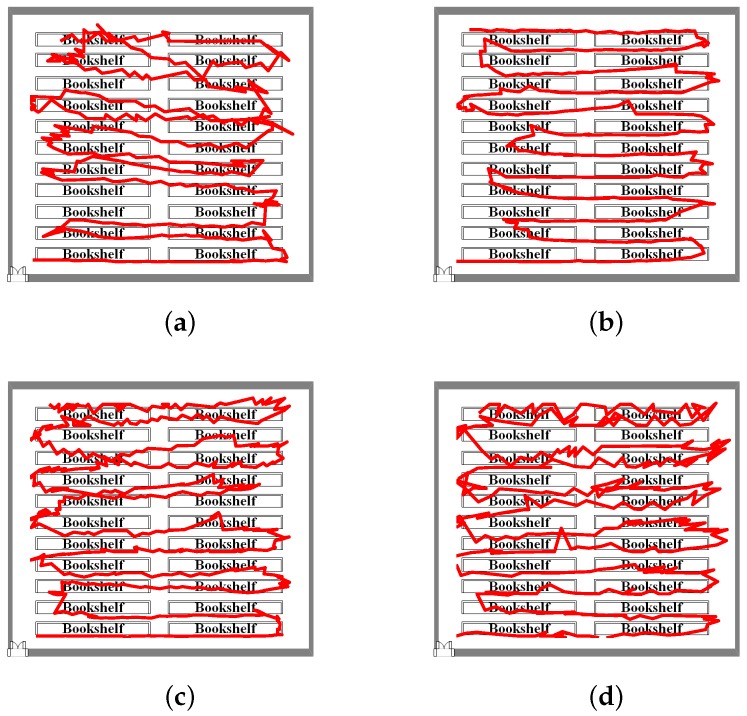
Positioning results with different gestures: (**a**) calling; (**b**) dangling; (**c**) handheld; and (**d**) pocket.

**Figure 13:**
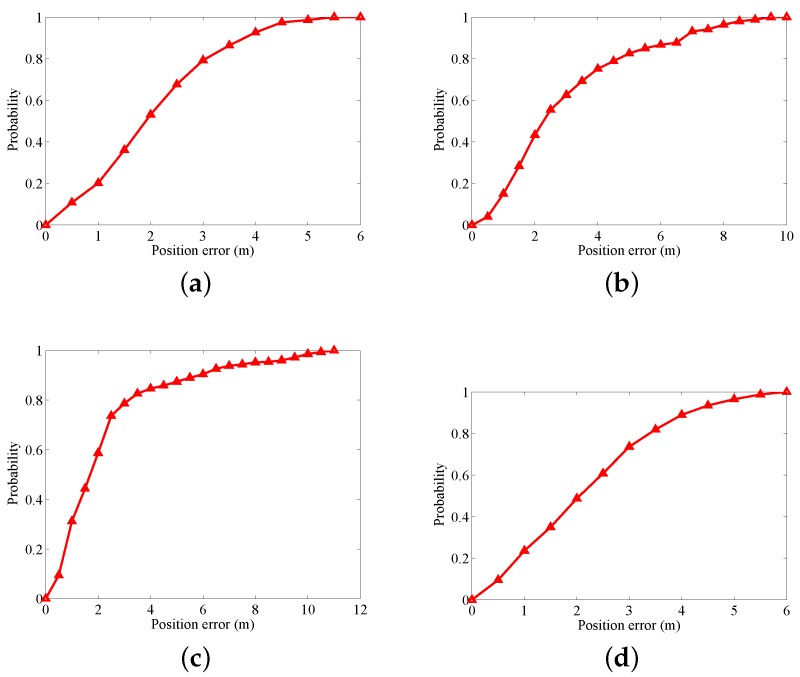
CDF with different gestures: (**a**) calling; (**b**) dangling; (**c**) handheld; and (**d**) Pocket.

A more complex walking experiment was conducted in a library stack room. The walking distance was about 260 m. Figure 12 shows the positioning results from library stack room walking experiment. When the position error is less than three meters, position error probability for calling, dangling, handheld and pocket gestures are 20.74%, 37.44%, 21.36% and 26.38%, respectively (see Figure 13). By using existing WiFi APs, the fusion positioning system based on WLS reduces position errors. Experimental results show that, for the four gestures, the positioning error is relatively stable.

To further verify the stability of the system, six volunteers participated in the walking experiment. Table 3 shows the positioning errors for the four motion gestures. Overall, for the six volunteers, the average error, RMSE and maximum error are as follows: (a) Person 1: 2.4 m, 2.98 m, and 7.83 m, respectively; (b) Person 2: 4.01 m, 5 m, and 11.99 m, respectively; (c) Person 3: 3.16 m, 3.98 m, and 11.02 m, respectively; (d) Person 4: 3.54 m, 4.39 m, and 10.77 m, respectively; (e) Person 5: 3.87 m, 4.76 m, and 10.78 m, respectively; and (f) Person 6: 3.96 m, 4.89 m, and 12.3 m, respectively. For users of different height, weight, and age, the INS/WiFi hybrid system estimates the location of the user very well. The localization results for different users show that the INS/WiFi hybrid positioning system is highly robust.

## 5. Conclusions and Future Work

INS errors accumulate with time. Complex and changeable environments disturb WiFi fingerprints. Therefore, it is difficult for a single-source positioning system to provide reliable indoor positioning. In this paper, a hybrid positioning system was proposed. First, an angle estimation model and a step length model were introduced. Second, the pre-processing mechanisms were used to improve radio signal quality. A threshold-based detection mechanism was used to remove unreliable RSSs. An Adaptive interpolation mechanism enriches the wireless signals. Third, because WiFi sequence matching is a multi-dimensional fingerprint matching, we accordingly proposed a MDTW to calculate the distance between the collected wireless signals and the WiFi fingerprints. To improve positioning accuracy and robustness, a MDTW-based WLS was proposed. By minimizing the square of the error, the hybrid system effectively reduced position errors.

Using four motion gestures, we conducted three walking experiments in the corridor, study room, and library stack room. Their average errors are 1.99, 1.71 and 2.4 m, respectively. In addition, RMSEs for three walking experiments are 2.41, 2.02 and 2.98 m, respectively. Comparing with the corridor walking experiment and the study room walking experiment, the worst positioning accuracy belongs to the library stack room walking experiment. Two main reasons led to the worst positioning accuracy. The first is a relatively long walking distance, resulting in a decrease in the positioning performance. The second is that a relatively small number of APs are used in the library stack room experiment (113 APs in the corridor, 80 APs in the study room, and 22 APs in the library stack room).

In future work, we will focus on how to further reduce the cumulative drift of INS and fluctuations in the wireless signals. More and more sensors are being integrated into smartphones. Therefore, to improve indoor positioning accuracy and robustness, future research will also focus on the integration of more sensors.

## Figures and Tables

**Table 1:** Position errors for INS, WiFi and INS/WiFi (m).

Motion Gestures	Error	The Average Error	RMSE	Maximum Error	CEP (95%)
**Calling**	INS	4.67	5.53	12.02	10.51
	WiFi	6.94	8.69	27.62	12.68
	INS/WiFi	1.93	2.23	4.55	3.92
**Dangling**	INS	3.61	4.36	9.54	9.08
	WiFi	5.79	9.62	33.73	33.72
	INS/WiFi	1.88	2.23	5.41	3.86
**Handheld**	INS	10.35	14.23	40.68	26.18
	WiFi	6.33	7	11.78	11.35
	INS/WiFi	1.48	1.72	3.75	3.26
**Pocket**	INS	5.35	6.35	12.13	11.05
	WiFi	18.24	20.22	31.32	30
	INS/WiFi	2.68	3.47	7.21	6.87
**General**	INS	4.72	5.55	10.81	9.9
	WiFi	9.33	11.38	26.11	21.94
	INS/WiFi	1.99	2.41	5.23	4.48

**Table 2:** Position errors for RADAR, MDTW, and WLS (m).

Motion Gestures	Error	The Average Error	RMSE	Maximum Error	CEP (95%)
**Calling**	RADAR	10.58	13.63	34.52	32.41
	MDTW	2.54	3.05	6.3	5.67
	WLS	1.74	2.05	4.37	3.82
**Dangling**	RADAR	5.47	8.64	28.41	26.53
	MDTW	2.05	2.38	4.96	4.04
	WLS	1.25	1.54	3.98	3.02
**Handheld**	RADAR	10.35	14.23	40.68	26.18
	MDTW	3.36	3.73	7.7	6.25
	WLS	1.97	2.31	5.4	4
**Pocket**	RADAR	27.44	29.97	50.81	46.09
	MDTW	2.23	2.51	5.08	4.02
	WLS	1.87	2.16	5.05	3.49
**General**	RADAR	13.46	16.62	38.61	32.8
	MDTW	2.55	2.92	6.01	5
	WLS	1.71	2.02	4.7	3.58

**Table 3:** Position errors with different persons (m).

Motion Gestures	Error	Person 1	Person 2	Person 3	Person 4	Person 5	Person 6
**Calling**	The average error	2.05	2.23	3.2	1.75	2.28	3.62
	RMSE	2.37	2.68	3.97	2.17	2.82	4.31
	Maximum error	5.21	6.38	10.01	5.41	6.88	9.48
**Dangling**	The average error	2.98	6.74	1.88	4.65	4.8	3.58
	RMSE	3.69	8.44	2.39	5.68	6.34	4.37
	Maximum error	9.46	20.22	6.79	11.52	15.22	10.89
**Handheld**	The average error	2.35	3.41	4.04	3.89	4.54	3.81
	RMSE	3.27	4.02	4.79	4.78	5.37	5.08
	Maximum error	10.7	8.44	11.64	11.93	10.59	15.04
**Pocket**	The average error	2.2	3.67	3.5	3.88	3.86	4.83
	RMSE	2.58	4.85	4.77	4.92	4.49	5.78
	Maximum error	5.95	12.92	15.62	14.23	10.43	13.8
**General**	The Average error	2.4	4.01	3.16	3.54	3.87	3.96
	RMSE	2.98	5	3.98	4.39	4.76	4.89
	Maximum error	7.83	11.99	11.02	10.77	10.78	12.3

## Abbreviations

The following abbreviations are used in this manuscript:

INS	Inertial Navigation System
MDTW	Multi-dimensional Dynamic Time Warping
WLS	Weighted Least Squares
GPS	Global Positioning System
BDS	Beidou Navigation Satellite System
MEMS	Micro-Electro-Mechanical System
APs	Access Points
RSS	Received Signal Strength
DTW	Dynamic Time Warping
HDE	Heuristic Drift Elimination
EKF	Extended Kalman Filter
APF	Auxiliary Particle Filter
KF	Kalman Filter
DR	Dead-Reckoning
MM	Magnetic Matching
DCM	Direction Cosine Matrix
CDF	Cumulative Distribution Function
REMS	Root Mean Square Error
CEP	Circular Error Probability
